# Supplementary material for: Figeno: multi-region genomic figures with long-read support
Source: Bioinformatics. 2024 Jun 10;40(6):btae354. doi: 10.1093/bioinformatics/btae354 (PMC11184262; doi:10.1093/bioinformatics/btae354)
Supplement: btae354_Supplementary_Data [file btae354_supplementary_data.pdf]

Supplementary information

Figeno: multi-region genomic figures with long-read support

Etienne Sollier[1,2], Jessica Heilmann[1], Clarissa Gerhäuser[1],
Michael Scherer[1], Christoph Plass[1], Pavlo Lutsik[1,3]

[1]Division of Cancer Epigenomics, German Cancer Research Center (DKFZ), Heidelberg, Germany

[2]Faculty of Biosciences, Ruprecht-Karls-University of Heidelberg, Heidelberg, Germany

[3]Department of Oncology, KU Leuven, Leuven, Belgium

Supplementary Table 1: Feature comparison of genomic visualization software in comparison to figeno.

	Figeno	IGV	Trackplot	Gviz	PyGenome Tracks	NeoLoop Finder	Methplotlib	Methylartist
Programming language	Python	Java	R	R	Python	Python	Python	Python
Multi-region support	✔	✔	✘	✘	✘	✔	✘	✘
Bigwig	✔	✔	✔	✔	✔	✔	✘	✘
HiC	✔	✘	✘	✘	✔	✔	✘	✘
Reads with base modification	✔	✔	✘	✘	✘	✘	✔	✔
Base modification frequency	✔	✘	✘	✔	✘	✘	✔	✔
WGS (copy number, SV)	✔	✘	✘	✘	✘	✘	✘	✘
Graphical user interface	✔	✔	✘	✘	✘	✘	✘	✘
Interactive exploration	✘	✔	✘	✘	✘	✘	✘	✘
Vector graphics export	✔	—*	✔	✔	✔	✔	✔	✔

*IGV can export to svg, but the resulting files are usually very large and difficult to edit further.

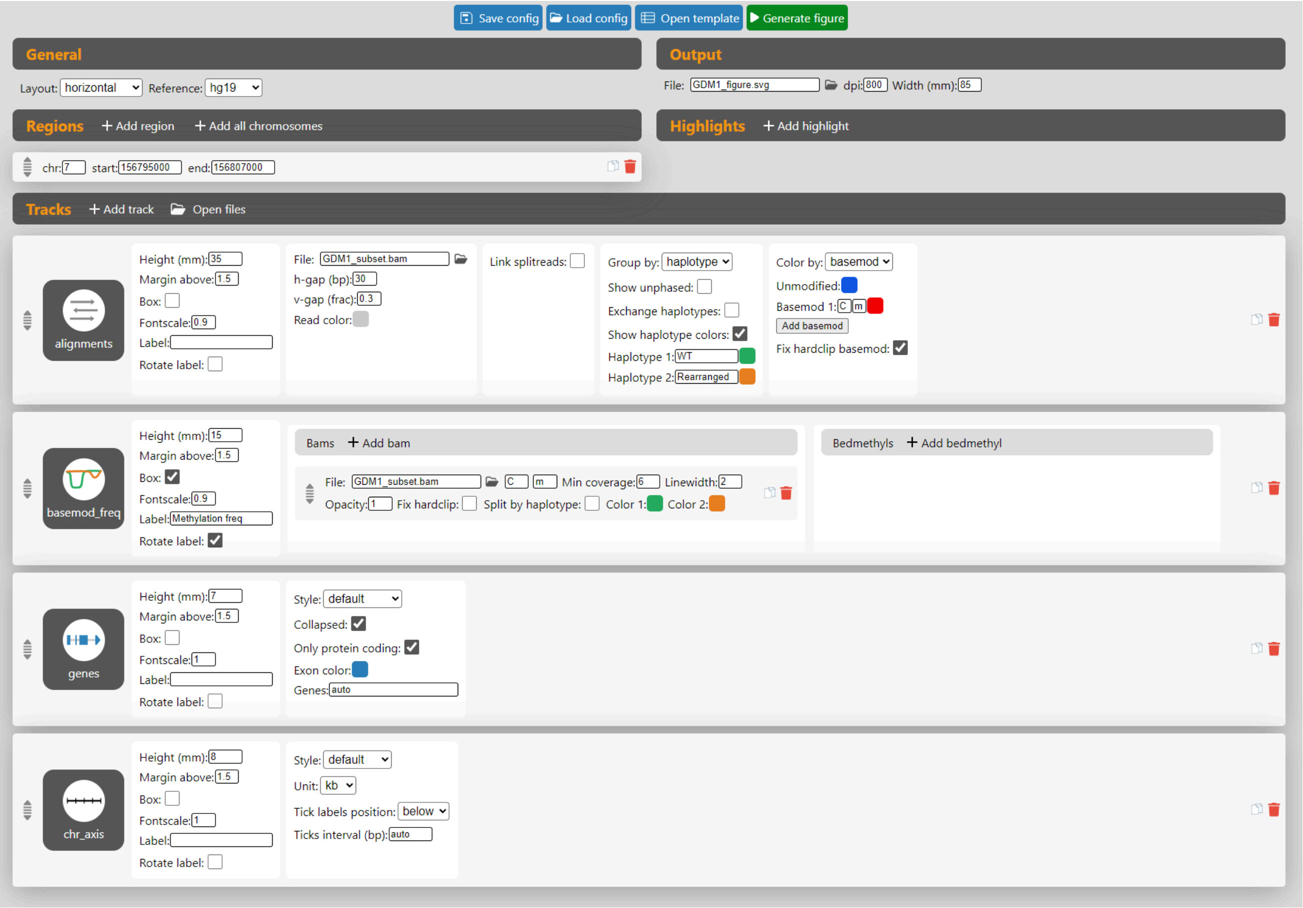

Supplementary Figure 1: graphical user interface. Screenshot of figeno's graphical user interface, here with the configuration for generating the allele-specific methylation plot for GDM-1 (Fig. 1D), with one region and four tracks: alignments, basemod_freq, genes and chr_axis.

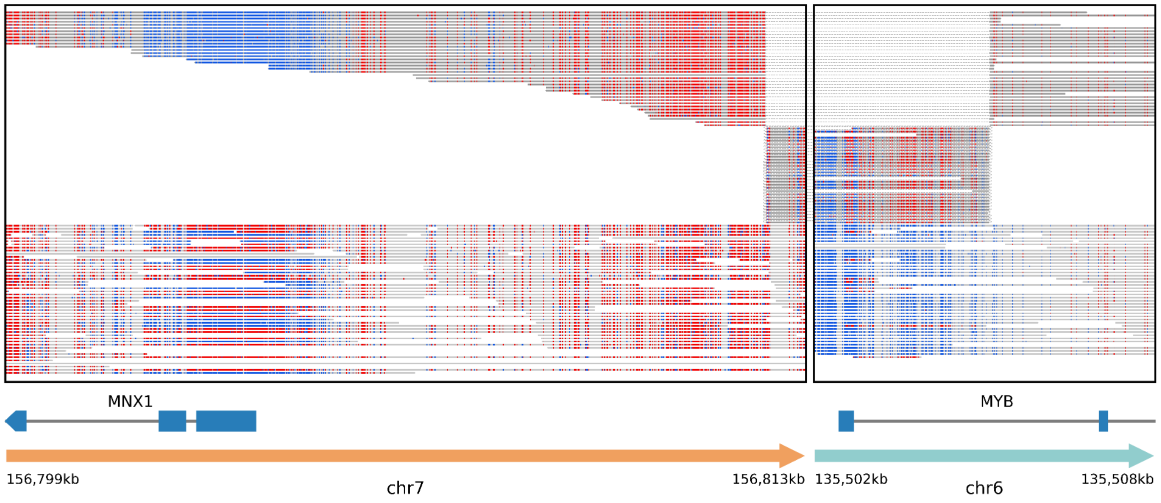

Supplementary Figure 2: split-read visualization. Nanopore reads for the GDM-1 cell line around the t(6;7) breakpoints. Alignments coming from the same read are linked together by dashed lines.

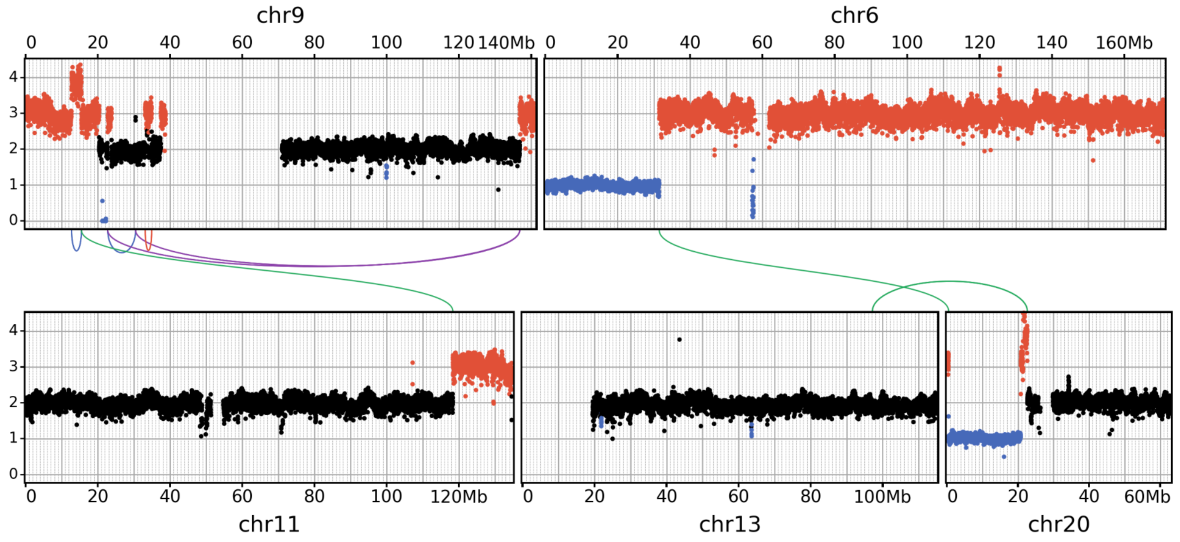

Supplementary Figure 3: symmetrical layout. Copy number and structural variants for chromosomes 6, 9, 11, 13, and 20 for the THP-1 cell line (whole genome sequencing data from the cancer cell line encyclopedia), visualized with figeno's symmetrical layout.
